# Quantum oscillations turn log(*B* )-periodic in Dirac semimetals: ‘Who ordered that?’

**DOI:** 10.1093/nsr/nwz016

**Published:** 2019-01-30

**Authors:** Ziqiang Wang

**Affiliations:** Department of Physics, Boston College, USA

**Figure 1.**
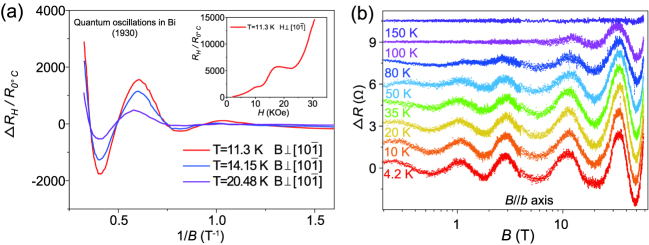
Quantum oscillations in magnetoresistance (MR). (a) Shubnikov–de Haas (SdH) oscillations in bulk single crystals of Bi after subtracting a polynomial background from the raw data. The inset shows a typical MR curve at 11.3 K. (b) Log(*B*)-periodic quantum oscillations in ZrTe_5_ after subtracting a smooth background. The raw data of MR in (a) are adapted from [1–3], and those in (b) from [4].

Quantum oscillations refer to the oscillatory behavior of transport and thermodynamic quantities as a function of the applied magnetic field *B* in metals and semimetals. The primary examples are the Shubnikov–de Haas (SdH) oscillations in the magnetoresistance *R*(*B*) [1] and the de Haas–Van Alphen effect in magnetization [2], both originally discovered in bulk single crystals of Bi in 1930. They are among the first macroscopic quantum effects observed and understood by mankind, based on Landau's quantization of a charged particle in a magnetic field in the same year [3]. These oscillations are periodic in 1/*B* (see Fig. 1a) and come from the oscillations in the density of states at the Fermi level *N*(*E*_F_) due to the arithmetic progression of quantized Landau levels. Over the past 90 years or so, quantum oscillations have served as a powerful experimental technique for probing the low-energy electronic properties of crystal solids.

A remarkable discovery was made recently by a team of researchers who reported magnetoresistance oscillations (see Fig. 1b) that are periodic in log(*B*), NOT in 1/*B*, in high-quality single-crystal ZrTe_5_, a 3D topological Dirac semimetal [4]. The carrier density in their ZrTe_5_ crystals is so low that a magnetic field *B* > 0.2 T already drives the sample into the quantum limit where only the lowest Landau level is occupied, and the 1/*B*-periodic oscillations must terminate. In high-quality 2D semiconductor heterostructures, increasing *B* further into the extreme quantum limit turns the quantum oscillations of the integer quantum Hall effect [5] into brand new ones associated with the fractional quantum Hall effect (FQHE) [6], which originates from the strong electron–electron interaction and disorder, but that are without clearly defined periodicity. In the extreme quantum limit of ZrTe_5_ for *B* > 0.2 T, the team observed five complete oscillations periodic in log(*B*) for *B* up to 58 T (Fig. 1b), where the filling fraction of the lowest Landau level would be smaller than 1/100! More spectacular is that these log(*B*) periodic quantum oscillations survive temperatures as high as 100 K! The authors ruled out speculations of FQHE, Wigner crystallization, and other symmetry-breaking density-wave orders as possible origins. The discovered log(*B*)-periodic oscillations constitute only the third known distinct type of periodicity in the nearly 90 years of quantum oscillation measurements in solids, with the second one being the Aharonov–Bohm effect that is periodic in *B*. Hence, there is a true moment of ‘Who ordered that?’, quoting I. I. Rabi on the occasion of the discovery of the unexpected muon.

On the plot of *R*(*B*) versus log(*B*), if we label either the minima or the maxima of the periodic oscillations by *B_n_*, the finding amounts to a geometric series *B_n_*/*B_n_*_+1_ = λ, where λ is a constant of order unity, around 3 in the ZrTe_5_ samples studied [4]. Naturally, one can think of the Fermi level density of states *N*(*E*_F_) acquiring such an oscillation, which in turn suggests that *N*(*E*) is modulated by the presence of a geometric sequence of quasi-bound states or resonances at energies *E_n_*/*E_n_*_+1_ = constant, reflecting an underlying discrete scale invariance (DSI). The authors consider the known example of DSI, the Efimov three-body bound states extensively studied in nuclear physics and recently observed in cold atom systems, to be unlikely since the resonant scattering condition is difficult to materialize over the wide range of magnetic fields. Instead, it is conjectured and supported by theoretical calculations that, owing to the relativistic energy–momentum dispersion relation in the Dirac semimetal, two-body quasi-bound states can emerge at charged impurity centers from the Coulomb interaction. Pictorially, the Coulomb impurities capture the Dirac quasiparticles to form artificial ‘atoms’ with quasi-bound state energies *E_n_* satisfying the geometric progression and DSI when the magnetic length becomes shorter than the screening length and plays the role of the cutoff for the wavefunctions. A subsequent theoretical paper [7] has shown more comprehensively that the log(*B*) periodic oscillations in the magnetoresistance arise from the scattering of electrons off the impurities with enhanced cross-sections at these energies, and are consistent with the existing data obtained in both the Dirac and Weyl semimetals ZrTe_5_ and TaAs respectively with different values of λ.

Since impurities are the rule rather than the exception, the discovered log(*B*)-periodic quantum oscillations are fingerprints of 3D Dirac and Weyl semimetals in the extreme quantum limit. Indeed, very recently, log(*B*)-periodic quantum oscillations in both magnetoresistance and Hall traces have been observed in another topological material, HfTe_5_ crystal [8]. It remains to be seen how the non-trivial topological properties in these materials, such as those responsible for the chiral anomaly and topological surface states, play out in the quantum oscillations. While further understanding of the origin and universality is necessary, an immediate implication of these findings is that the log(*B*)-periodic oscillations should be observable in other transport and thermodynamic properties of topological semimetals, which are awaiting discovery.
